# Superconducting Carbon‐Cage Network with *T*_*c*_ of 109 K at Ambient Pressure

**DOI:** 10.1002/advs.202504281

**Published:** 2025-05-29

**Authors:** Z. F. Ye, David J. Singh, Y. N. Huang, Guo‐Hua Zhong, Hai‐Qing Lin

**Affiliations:** ^1^ Department of Physics Zhejiang University of Science and Technology Hangzhou 310023 China; ^2^ Department of Physics and Astronomy University of Missouri, Columbia Missouri 65211‐7010 USA; ^3^ Shenzhen Institutes of Advanced Technology Chinese Academy of Sciences Shenzhen 518055 China; ^4^ School of Physics Zhejiang University Hangzhou 310058 China

**Keywords:** cage‐network, carbon‐based materials, density functional theory, eliashberg theory, high‐temperature superconductors

## Abstract

A novel carbon‐cage network was reported, denoted as C_18_, found to be a low energy structure by first principles particle swarm structure search. The compound exhibits high temperature electron–phonon ambient pressure superconductivity with *T*_*c*_ = 79 K for elemental doping and can be raised to *T*_*c*_ = 109 K by appropriate hole doping. Analyses of the phonon spectra, molecular dynamics simulations, and enthalpy differences relative to analogous structures synthesized experimentally all suggest that the hole‐doped high‐*T*_*c*_ structure is a viable candidate for experimental synthesis.

## Graphical abstract

By introducing elemental and hole doping into a theoretically designed sp3‐hybridized C_1_8 carbon cage, superconductivity with a transition temperature up to 109 K is predicted under ambient pressure. These findings offer a promising pathway for designing high‐Tc superconductors based on stable covalent carbon frameworks.

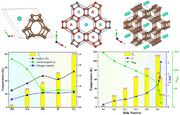

## 1 Introduction

Superconductivity, especially with high critical temperature(*T*_*c*_) is much sought after in condensed matter physics. Hydrides^[^ ^1^, ^2^, ^3^, ^4^, ^5^, ^6^ ^]^ have shown electron‐phonon superconductivity close to room temperature at high pressure, offering new directions in the study of high‐T_*c*_ superconductors. However, the instability of these materials at low pressure has limited their potential, thereby stimulating the exploration of non‐hydrogen containing superconducting materials,^[^ ^7^, ^8^ ^]^ which may exhibit high‐*T*_*c*_ superconductivity at ambient pressure. Carbon‐based materials, characterized by their chemical robustness and diverse crystalline configurations, offer one attractive possibility. Graphene based superconducting materials can form layered structures by intercalating metal atoms between graphene layers, for example, YbC_6_ and CaC_6_^[^ ^9^, ^10^, ^11^ ^]^ exhibit superconductivity at 6.5 and 11.5 K, respectively. Magic‐angle graphene^[^ ^12^, ^13^, ^14^ ^]^ exhibits hole‐doped superconductivity by forming a twist angle between graphene layers. In addition, various theoretical proposals have been made.^[^ ^15^, ^16^, ^17^, ^18^, ^19^, ^20^, ^21^ ^]^ Among pure carbon cage superconducting materials, the most well‐known is fullerene (C_60_), whose *T*_*c*_ can be tuned to a range of 18 − 40 K through alkali metal doping.^[^ ^22^, ^23^, ^24^, ^25^ ^]^ Recent theoretical work suggests that appropriately doped small cage, C_24_ and C_32_ based structure may be particularly promising.^[^ ^26^, ^27^ ^]^ A key issue is the realizability of predicted superconducting materials. For this, reasonably low energy and at least dynamic stability are needed. In the case of carbon, the strong directional bonds favor the possibility of forming many allotropes, as observed nature.

Here, we employed the CALYPSO^[^ ^28^ ^]^ algorithm to predict a novel C_18_ cage network, and subsequently utilized first‐principles calculations to assess the impact of elemental and hole doping on its superconducting properties under ambient conditions. Although the CALYPSO^[^ ^28^ ^]^ search identified a low energy C_18_ structure, further examination of the structure showed that it has an instability against a distortion. This instability is removed when the electron count is reduced to the semiconducting and hole doped compositions. As such we focus on the holed‐doped compositions in this manuscript. We use Bardeen‐Cooper‐Schrieffer (BCS)^[^ ^29^ ^]^ and Migdal‐Eliashberg theories^[^ ^30^ ^]^, to evaluate the superconductivity in the C_18_ lattice. We find robust electron‐phonon coupling (EPC), high phonon frequencies, and a substantial density of states (DOS) at the Fermi level (*E*_*F*_). We investigated the phonon dynamics, electronic structures, and doping effects to find the mechanisms that enhance superconductivity, specifically by strengthening the EPC and increasing the DOS at *E*_*F*_. Doping analysis of CsC_12_ reveals a high‐*T*_*c*_ at 79 K. Hole doping of 0.6 per unit cell further enhances *T*_*c*_ to 109 K, attributed to elevated DOS at the *E*_*F*_ and enhanced EPC.

## 2 Results

### 2.1 Element Doping

**Figure 1:**
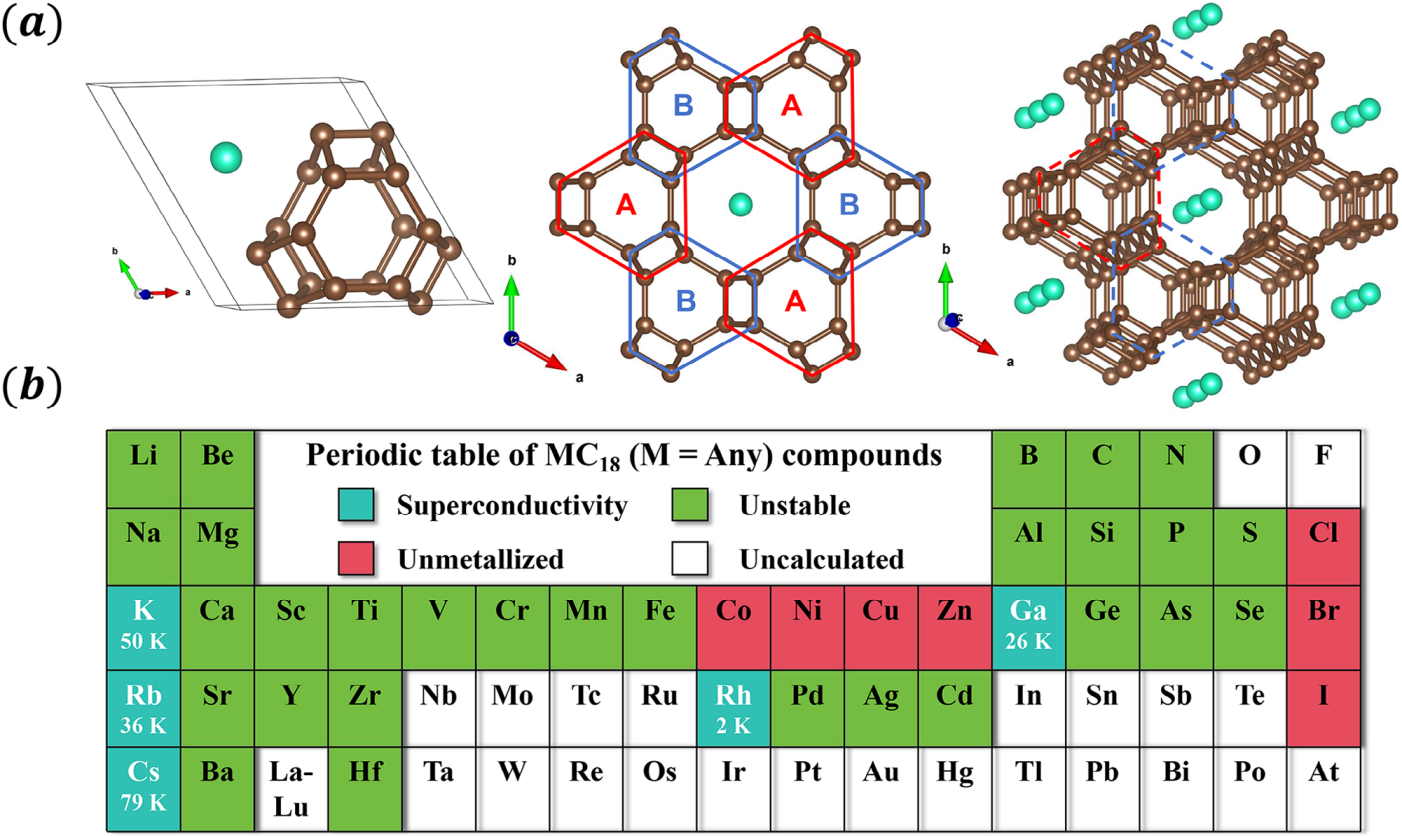
a) Schematic diagram of metal‐doped carbon cage network, including *M*C_12_ unit cell, stacking method and 3D spatial layout. b) The periodic table of *M*C_12_ compounds. Cyan indicates stability and superconductivity, red indicates a stable structure but no superconductivity, green indicates an unstable structure, and white indicates not calculated.

The study investigates the metal doped C_18_ cage network with the stoichiometry of *M*C_12_ (*M* represents the dopant element), which is depicted in **Figure** **1**a and crystallizes in the *Amm*2 space group (No. 38), characterized by a unique tetrahedral coordination of 18 carbon atoms. This carbon‐cage network, with a coordination number of four, has structural attributes related to the diamond lattice,^[^ ^31^, ^32^, ^33^, ^34^ ^]^ as seen in its 3D atomic arrangement. The *M*C_12_ cage network is structured into three distinct segments: an upper layer, a middle layer, and a lower layer. The upper and lower regions each consist of two regular hexagonal arrays, whereas the middle region presents a hexagon with a non‐uniform geometry. To delineate the stacking architecture, the middle regions of two contiguous carbon‐cages are designated as A(red) and B(blue), respectively. The interlayer offset, distinguishing A from B, is quantified by a displacement of *c*/2. The stacking sequence of the *M*C_12_ crystal structure adheres to an ABABAB… pattern, forming the lattice. Within this framework, the dopant is uniquely positioned outside the carbon‐cage network, occupying the cyan atom sites as depicted in Figure 1a, coexisting with the red A middle layer and contributing to stabilizing this configuration. We examined the impact of various dopant elements on the metallization and superconducting properties of the *M*C_12_ structure across the periodic table, compiling our findings into an *M*C_12_ metallization periodic table as depicted in Figure 1b. Our analysis reveals that only the cyan‐highlighted elements, including K, Rb, Cs, Ga, and Rh, are capable of inducing metallization and superconductivity in the *M*C_12_ structure under ambient conditions, with CsC_12_ achieving the highest *T*_*c*_.

The electron–phonon interaction is a critical mechanism for superconductivity. Our work focuses on examining the phonon properties and electronic configurations of dopants to understand their influence on electron–phonon coupling and the associated impact on *T*_*c*_. The bonding Electron Localization Function (ELF) illustrated in the Figure S1 (Supporting Information) is utilized to analyze the chemical bonding. Specifically, for the *M*C_12_ structure doped with alkali metals Cs, Rb, and K, the ELF values of C‐C bonds are all above 0.9, indicating the formation of strong covalent bonds via *sp* ^3^ hybridization. Comparisons were made with graphene, C‐P6_3_mmc (*sp* ^2^ hybridization) and diamond (*sp* ^3^ hybridization), as shown in Figure S2 (Supporting Information). Such covalent bonds are crucial for the structural stability of the material, maintaining its stability at ambient pressure. Additionally, the alkali metals exhibit ionic bonds. Conversely, the ELF mappings for the remaining two structures exhibit discernible disparities, particularly within the hexagonal C‐C bond domains. Within these regions, changes in the ELF intensity are noted, with a concomitant reduction in ELF values in the proximity of dopant atoms, indicative of a diminished bond strength. In conclusion, the robust nonpolar covalent interactions within the C_18_ cage framework, characterized by elevated ELF values in the vicinity of dopant atoms, reinforce the electronic bonding integrity.

**Figure 2:**
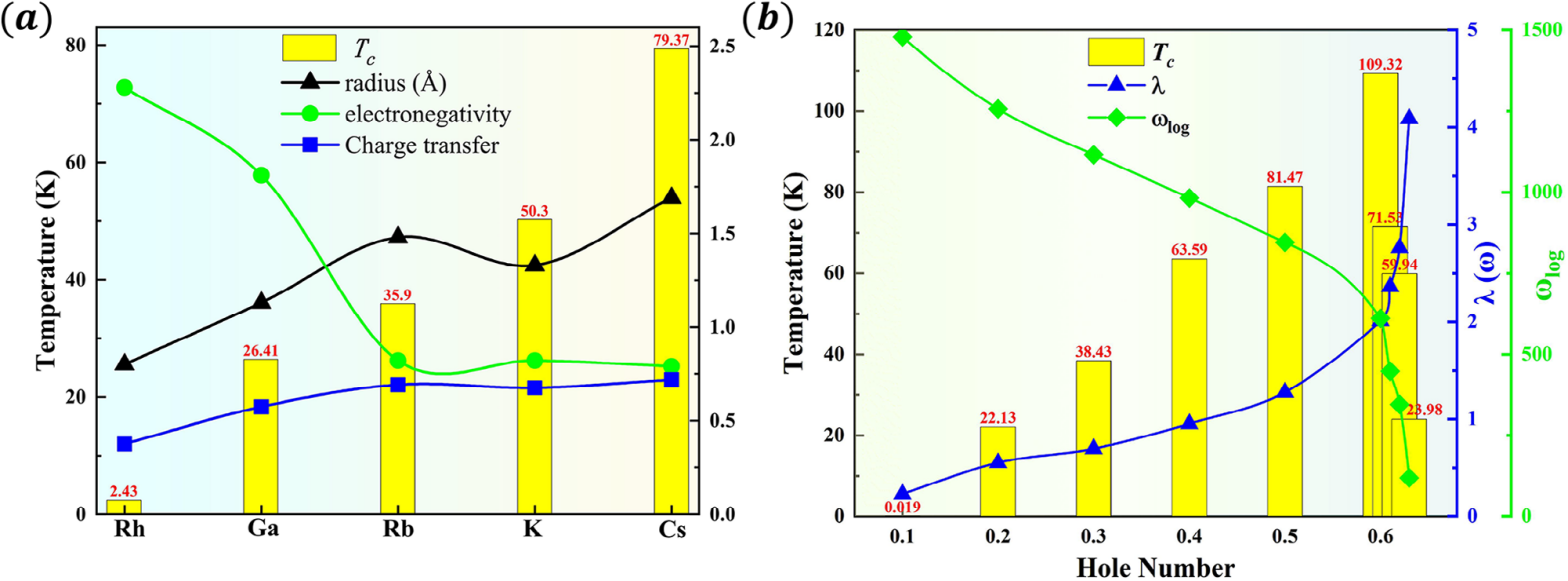
a) The influence of ionic radius, electronegativity and charge transfer of doping elements on *T*_*c*_. b) The relationship between hole doping concentration *T*_*c*_ and EPC constant λ(ω) and logarithmic mean frequency ω_*log*_.

We investigated the impact of dopants' ionic radii, electronegativities, and charge transfer on the *T*_*c*_, as depicted in **Figure** **2**a. Notably, the observed *T*_*c*_ trend in MC_12_ (Cs >K >Rb) contrasts with the A_3_C_60_ system (Cs_3_C_60_ >Rb_3_C_60_ >K_3_C_60_),^[^ ^35^, ^36^ ^]^ where lattice expansion dominates. This divergence highlights the distinct EPC mechanisms in our 3D sp^3^‐bonded framework compared to molecular fullerides. To better understand this, Bader charge^[^ ^37^, ^38^ ^]^ analysis was performed to quantify the amount of charge transferred from each alkali metal dopant. The results indicate that Cs donates the most charge (0.72 *e*/Cs), while K and Rb exhibit comparable values (0.675 *e*/K and 0.69 *e*/Rb), consistent with their respective electronegativities. We further estimated the charge carrier density by considering the unit cell volumes. Due to the smaller ionic radius of K, the unit cell volume of KC_12_ (91.5 Å^3^) is slightly smaller than that of RbC_12_ (93.7 Å^3^), resulting in a marginally higher carrier density for KC_12_. This may partially account for its higher *T*_*c*_ compared to RbC_12_. We would like to emphasize that carrier density alone is not sufficient to fully explain the observed trends. As summarized in **Table** **1**, CsC_12_ despite having the lowest carrier density exhibits the highest *T*_*c*_. This indicates that the dominant contributors to *T*_*c*_ in alkali‐metal‐doped C_12_ frameworks are the EPC strength and the DOS near the Fermi level. The enhanced λ and DOS in CsC_12_ are primarily responsible for its superior superconducting performance.

**Table 1:** Calculated electron‐phonon coupling constant (λ), electronic density of states at the Fermi level (DOS), unit cell volume, and charge carrier density for MC_12_ (M = K, Rb, Cs) cage structures.

Parameter	CsC_12_	RbC_12_	KC_12_
λ	1.9504	0.8565	0.9498
DOS(states/eV)	2.52	1.48	1.90
Volume (Å^3^)	100.2	93.7	91.5
Charge Density(10^21^ecm^−3^)	7.156	7.356	7.377

**Figure 3:**
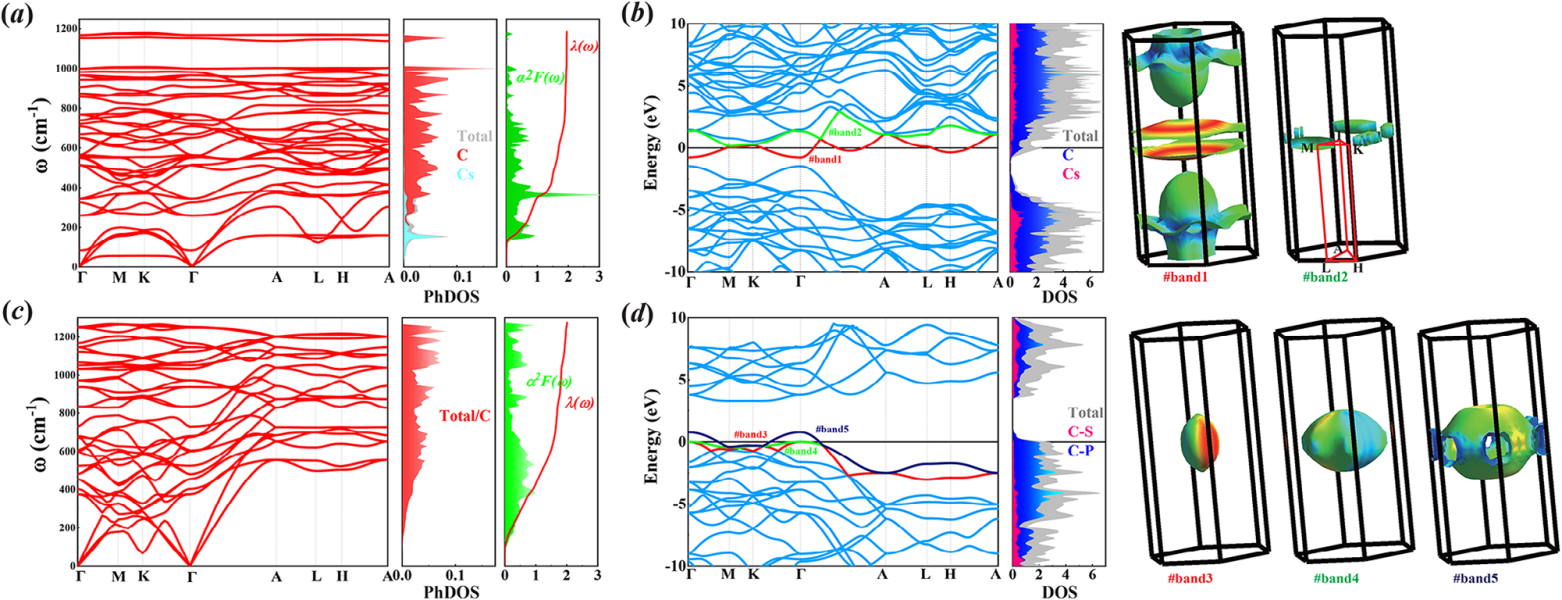
Phonon properties and electronic structure of CsC_12_ cage network and 0.6 hole‐doped C_18_ cage network at 0‐GPa, (a, c) phonon spectrum, PhDOS, Eliashberg spectral function and EPC integral, (b, d) band structure, DOS and Fermi surfaces.

The CsC_12_ cage network, noted for its elevated *T*_*c*_, serves as a model system in our investigation to unravel the factors contributing to its enhanced *T*_*c*_. First, we studied the phonon characteristics of CsC_12_. **Figure** **3**a presents the phonon spectrum, element‐projected phonon density of states (PhDOS), Eliashberg spectral function (α^2^ *F*(ω)), and EPC integral λ(ω) along high‐symmetry paths for CsC_12_. The absence of negative frequencies within the phonon dispersion curves confirms the dynamic stability of CsC_12_ at ambient conditions. We also did calculations for the phonon dispersions of the undoped cage‐type carbide. This pristine form exhibits dynamic stability and is electronic insulating. The corresponding phonon dispersion relations are presented in Figure S2 (Supporting Information). Compared to cage‐type hydrides,^[^ ^39^, ^40^, ^41^ ^]^ the phonon frequencies of cage‐type carbides are much lower, corresponding to the heavier mass, with a maximum value of 1400 cm^−1^. Doping elements weaken the lattice vibrations and lower the phonon frequencies, with CsC_12_ having a maximum frequency of 1180 cm^−1^. The incorporation of heavier atoms induces significant phonon softening in the low‐frequency regime. Analysis of the element‐PhDOS reveals a pronounced peak attributed to Cs atoms around 150 cm^−1^, whereas the lighter C atoms exhibit peaks in the higher frequency domain. Additionally, due to the electron transfer from Cs atoms to C atoms, there is partial overlap of the PhDOS of C and Cs in the 200 − 370 cm^−1^ range. Cs contributes a relatively minor fraction to the overall electron–phonon coupling constant λ, specifically accounting for approximately 14.7%. In contrast, C atoms contribute significantly, comprising 85.3% of the total. This highlights the critical role of the carbon lattice vibrations in the superconductivity. By integrating α^2^ *F*(ω), we find that the EPC constant λ and the logarithmic average frequency ω_*log*_ are equal to 1.95 and 447.9 K, respectively. The elevated λ, in contrast to alkali‐doped C_60_^[^ ^42^, ^43^ ^]^ and B–C compounds,^[^ ^44^ ^]^ is attributed to the *sp* ^3^‐bonded framework and increased DOS at *E*_*F*_. Using the modified McMillian‐Allen‐Dynes^[^ ^45^ ^]^ formula with a typicalCoulomb potential μ^⋆^ = 0.1,^[^ ^45^, ^46^, ^47^ ^]^ we calculated *T*_*c*_ = 79 K(μ^⋆^ = 0.1, T_*c*_ = 71.8 K) for Cs‐doped C_18_ cage network, which is in the liquid nitrogen temperature range. The phonon characteristics of alternative structures are detailed in the Figure S4 (Supporting Information). Our analysis reveals a correlation between the elevation of the *T*_*c*_ following doping and a concomitant reduction in phonon frequencies. Specifically, the softened phonon modes in the low‐frequency domain are diminished, leading to an enhanced DOS in this region. Thus, this is a strong coupling superconductor that has a high *T*c due both to the large λ associated with phonon modulation of C‐C bonds and the high frequencies of those phonon reflected in ω_*log*_.

Figure 3b presents the band structure, element‐projected density of states, and Fermi surfaces of CsC_12_, elucidating the electronic properties of this material. Near the *E*_*F*_, there are mainly two bands (red #1 and green #2) crossing the *E*_*F*_, forming complex Fermi surfaces near the Γ point and the *M* − *K* path, respectively. The integrated contribution at *E*_*F*_ in CsC_12_ amounts to a substantial 2.52 states/eV, with C contributing significantly at 1.04 states/eV and Cs contributing a modest 0.13 states/eV The remaining 1.35 states/eV is attributed to the interstitial electrons, which are identified as the dominant contributors at *E*_*F*_. The findings indicate that electrons localized within the interstitial regions of the carbon framework play a crucial role in determining the *T*_*c*_. Figure S5 (Supporting Information) provides a detailed analysis of the band structure and DOS for alternative configurations. Notably, alkali metal doping exhibits characteristic features of electron doping, as reflected in similar traits within the band structure and distinct peaks in the DOS. The upward displacement of the *E*_*F*_ intersects with the conduction band, leading to the formation of electron‐pockets at the Fermi surface. A decrease in the size of these Fermi pockets is observed with a reduction in the radius of the dopant atoms. Furthermore, the enhancement of the DOS at the *E*_*F*_ correlates with the increase in *T*_*c*_. For example, CsC_12_ shows a peak in the DOS, while the *E*_*F*_ in KC_12_ and RbC_12_ shifts further upward, leading to a decrease in *T*_*c*_. The arrows represent the extent of the *E*_*F*_ shift, see Figure S6 (Supporting Information). Furthermore, for GaC_12_ and RhC_12_, the band structures show that each has a valence band crossing the *E*_*F*_, distinctly different from the alkali metal doping systems. In this comparative analysis, GaC_12_ is characterized by a reduced DOS at the *E*_*F*_, whereas RhC_12_ exhibits an enhanced DOS of 2.9 states/eV, predominantly associated with Rh, which correlates with a reduced *T*_*c*_. These findings suggest that the realization of a high *T*_*c*_ is contingent upon both an elevated DOS at the *E*_*F*_ and a significant contribution from lighter constituent elements.

### 2.2 Hole Doped C_18_ Superconductivity

The electronic structure analysis of the pristine cage network indicates that the *E*_*F*_ is positioned within a 2.24 eV bandgap, as shown in Figure S3 (Supporting Information). Strongly asymmetric DOS distribution implies that hole doping could effectively shift *E*_*F*_ downward into the high‐DOS region. This inspires the possibility of enhancing superconducting performance by doping with hole‐type carriers to adjust the *E*_*F*_.

To simulate hole doping in the C_18_ cage structure, we employed the Jellium model,^[^ ^48^ ^]^ which offers a well established theoretical approximation analogous to experimental electrostatic gating techniques. Such techniques–particularly ionic liquid gating or electrostatic field‐effect gating–have been successfully applied to modulate carrier concentrations in low‐dimensional materials, including magic‐angle twisted bilayer graphene^[^ ^12^ ^]^ and monolayer transition metal dichalcogenides.^[^ ^49^ ^]^ The key advantage of the Jellium approach lies in its ability to introduce excess charge in a spatially uniform manner, without introducing chemical dopants or structural distortions, thus allowing us to isolate the intrinsic effects of hole doping on superconductivity. Under hole doping, the *E*_*F*_ shifts downward. Figure 2b illustrates that as the density of holes per unit cell escalates, the *T*_*c*_ gradually increases as expected. A maximum doping level of 0.6 holes per unit cell is feasible for the C_18_ cage network, while keeping dynamic stability. At this doping level, *T*_*c*_ attains its maximum value of 109 K. Enhancing the doping concentration further results in a pronounced decrease in *T*_*c*_. Analysis of the phonon spectrum reveals that upon surpassing 0.6 holes per unit cell, the emergence of imaginary frequencies occurs, which is indicative of a loss in structural dynamic stability, see Figure S7 (Supporting Information).

With hole doping of 0.6 holes per unite cell in the C_18_ cage crystal, the phonon properties and electronic structures are depicted in Figure 3c,d. As shown in Figure 3c, the phonon spectrum of the hole‐doped C_18_ cage crystal lacks any imaginary frequencies. In comparison with the undoped system, the introduction of hole doping substantially diminishes the phonon frequencies, a phenomenon that aligns with the trend observed in elemental doping, suggesting that the doping of carriers leads to a reduction in phonon frequencies. Under hole doping conditions, the highest phonon frequency in the C_18_ cage network is found to decrease to 1260 cm^−1^. As shown in Figure S7 (Supporting Information), within the low‐frequency regime, the emergence of phonon softening modes is observed along the *M* − *K* direction in the Brillouin zone, with the magnitude of softening becoming more pronounced as the hole concentration increases. This behavior is analogous to that observed in elemental electron doping, where higher concentrations of electron‐type carriers lead to enhanced phonon softening, which is conducive to elevating the *T*_*c*_. In the context of the hole‐doped C_18_ cage network, the PhDOS exhibits a notable reduction in the peak intensity within the high‐frequency domain relative to the undoped counterpart. Concurrently, there is a substantial enhancement in the spectral weight contribution in the mid‐frequency domain, which enhances λ. Specifically, the mid‐frequency domain emerges as the predominant contributor to λ, exhibiting a sharp increase within the 200 − 700 cm^−1^ range. Within this interval, the contribution from α^2^ *F*(ω) surpasses 61.5%. Upon integrating α^2^ *F*(ω), λ and ω_log_ are 2.01 and 611.13 K, respectively. With the effective Coulomb pseudopotential μ^⋆^ set to 0.1,^[^ ^45^, ^46^, ^47^ ^]^ the *T*_*c*_ for the hole‐doped C_18_ is estimated to be *T*_*c*_ = 109 K(μ^⋆^ = 0.1, *T*_*c*_ = 102 K). As depicted in Figure 2b, the increase in *T*_*c*_ arises from the interplay between λ and ωlog. Despite the decrease in the vibrational frequency ω_log_ with increasing hole concentration, λ exhibits a more pronounced inverse increase relative to ω_log_, thereby resulting in a marked enhancement of *T*_*c*_. The findings demonstrate that by modulating the EPC strength, the *T*_*c*_ can be effectively controlled.

The electronic structure changes caused by hole doping are shown in Figure 3d. The band structure analysis reveals a downward shift of the *E*_*F*_ by 0.8 eV relative to the pristine system, with three bands (#3, #4, and #5) intersecting *E*_*F*_, thereby generating three intricate Fermi surfaces in proximity to the Γ point. As shown in Figure S8 (Supporting Information), orbital‐resolved band analysis indicates that the contribution of C‐*s* orbitals to *E*_*F*_ is negligible, confined to a minor distribution near the Γ point. Bands #3 and #4 are predominantly constituted by C‐*p*_*x*/*y*_ orbitals, whereas band #5 is predominantly governed by C‐*p*_*z*_ orbitals. The DOS analysis demonstrates that hole doping repositions *E*_*F*_ to the initial peak in the low‐energy domain, with the DOS amounting to 2.68 states/eV, marking an increment of 0.16 states/eV over CsC_12_. The carbon atoms contribute 1.92 states/eV, which is nearly double that observed in the element‐doped system, while the contribution from interstitial electrons is markedly diminished. This underscores the potential to effectively enhance *T*_*c*_ by modulating the DOS at *E*_*F*_, offering significant insights for the development of materials with elevated *T*_*c*_.

### 2.3 Structural Stability

**Figure 4:**
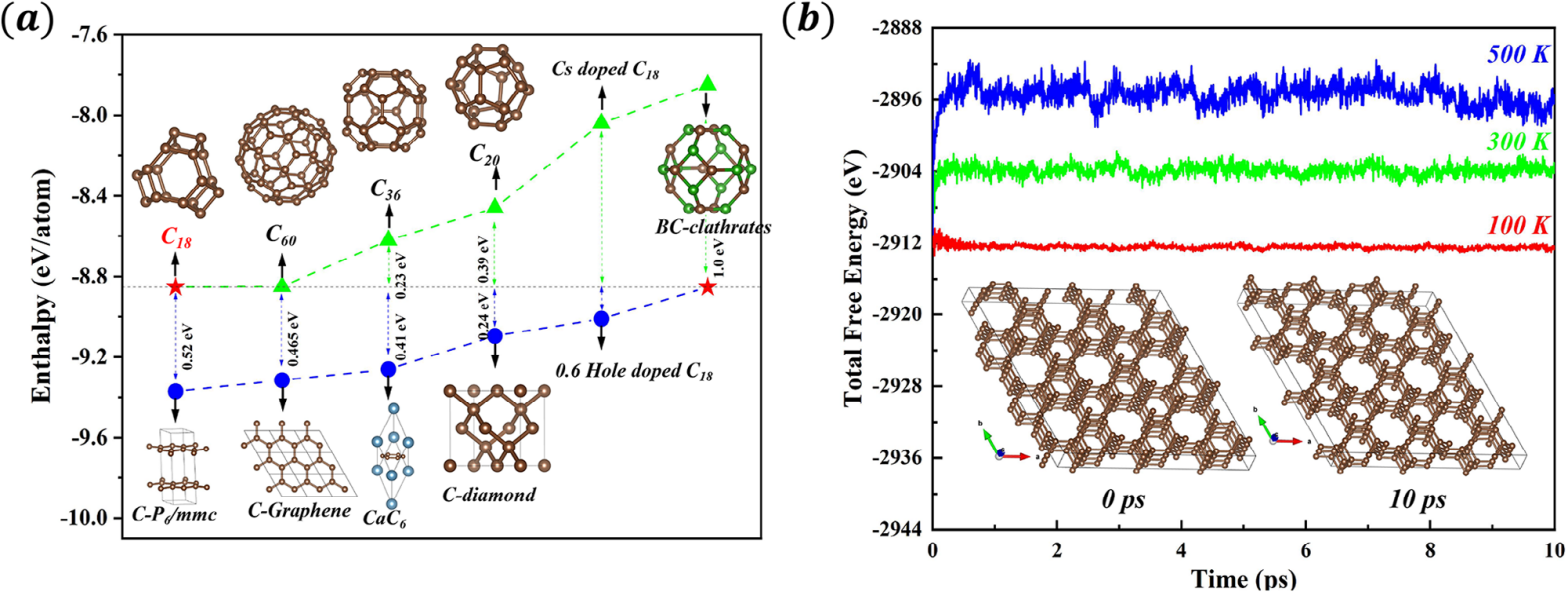
a) The enthalpy difference diagram of carbon‐based materials can be synthesized by experiment. b) Molecular dynamics simulation of a C_18_ cage network with 0.6 holes per unit cell.

In the final analysis, we address the experimental synthetic accessibility of the C_18_ cage network. **Figure** **4**a delineates the variations in atomic enthalpy values among diverse carbon allotropes. The C_18_ cage network is denoted by a red pentagram, while the green curve delineates the enthalpies of previously synthesized cage structures, including C_20_,^[^ ^50^, ^51^, ^52^ ^]^ C_36_,^[^ ^53^, ^54^, ^55^ ^]^ and C_60_,^[^ ^56^, ^57^, ^58^ ^]^ as well as the enthalpy of BC‐clathrates.^[^ ^44^, ^59^, ^60^ ^]^ The blue curve, in contrast, signifies the enthalpies associated with sheet‐like carbon materials^[^ ^61^, ^62^, ^63^ ^]^ and diamond structure.^[^ ^64^ ^]^ For CsC_12_, the formation enthalpy increases to −8.04 eV, slightly higher than that of C_20_ fullerene and comparable to reflecting a metastable configuration. The hole‐doped structure shows an even more favorable formation enthalpy, approaching that of diamond, indicating enhanced thermodynamic stability with doping. Although the enthalpy value of C_18_ does not exhibit a pronounced advantage over planar materials, it is comparatively lower than other cage‐like carbon structures. For instance, the atomic enthalpy of C_18_ is equivalent to that of C_60_, a carbon‐based closed‐cage molecule for which experimental synthesis techniques are well‐established. Furthermore, current synthetic methodologies have successfully yielded smaller carbon cage molecules, such as C_20_ and C_36_, with an enthalpy difference exceeding 0.2 eV when compared to C_18_. From a thermodynamic perspective, this suggests that the synthesis of C_18_ possesses certain merits. In recent years, the successful synthesis of SrB_3_C_3_ by Li et al., which exhibits a higher atomic enthalpy and a 1 eV enthalpy difference relative to C_18_, demonstrates that carbon‐based structures with elevated enthalpy can be synthesized under existing experimental conditions. Given that the enthalpy of C_18_ is equivalent to that of C_60_, its synthesis is relatively less challenging. See Figure S12 (Supporting Information), potential experimental methods include chemical vapor deposition (CVD), arc discharge, and template‐directed growth. These methods, successfully applied to synthesize C_60_ and carbon nanotubes, could be adapted for the C_18_ cage network by optimizing precursor design and confinement conditions.^[^ ^65^, ^66^ ^]^

This investigation employs NPT ensemble molecular dynamics simulations to assess the thermodynamic stability of the C_18_ cage network, with a particular emphasis on hole‐doped configurations possessing 0.6 holes per unit cell at ambient pressure. The simulation parameters encompass a cumulative simulation duration of 10 picoseconds, and temperature settings are specified at 100, 300, and 500 K to probe the thermodynamic response of the material in proximity to the *T*_*c*_, ambient temperature, and elevated temperature regimes, respectively. Figure 4b depicts the total energy fluctuations for the hole‐doped C_18_ cage network. Throughout the simulation, the energy fluctuations remained negligible, signifying the robust stability of the hole‐doped C_18_ cage network under the simulated conditions. Structural snapshots extracted from the simulation trajectory revealed that the atoms oscillated around their equilibrium positions, thereby corroborating the thermodynamic stability of the C_18_ cage network. Furthermore, phonon spectrum analysis confirmed the absence of imaginary frequencies within the C_18_ cage network, thereby attesting to its dynamic stability. By integrating the enthalpy diagram with molecular dynamics simulation results, the C_18_ cage network exhibits substantial thermodynamic and dynamic stability, with a significant enthalpy advantage over other synthetically accessible cage structures. These findings further reinforce the experimental viability of fabricating the C_18_ cage network, thereby laying a crucial foundation for the advancement of innovative carbon‐based superconducting materials.

## 3 Conclusion

This study has employed the CALYPSO software to predict a novel C_18_ cage network and conducted first‐principles calculations to explore its superconducting properties under elemental and hole doping at ambient pressure. Elemental doping has demonstrated a high‐*T*_*c*_ of 79 K for CsC_12_, while hole doping has achieved an even higher *T*_*c*_ of 109 K. Robust nonpolar covalent interactions within the C_18_ cage network ensure its structural stability under these conditions.

Our findings reveal that electronegativity and ionic radius of dopants significantly influence *T*_*c*_, primarily through their effects on unit cell volume, charge carrier density, and electron–phonon coupling strength. The uniform distribution of hole carriers has been shown to further improve EPC and facilitate Cooper pair formation. Moreover, the optimization of the DOS at the *E*_*F*_ has emerged as an effective strategy for enhancing *T*_*c*_.

Stability analyses confirm that the C_18_ cage network is dynamically and thermodynamically viable, with an enthalpy advantage over other carbon cage configurations, supporting its experimental realization. While our theoretical predictions provide a robust foundation, experimental validation remains critical. Future efforts should focus on synthesizing the C_18_ cage network using techniques such as laser vaporization, arc‐discharge methods or CVD, followed by characterization of its superconducting properties under doping.

## 4 Experimental Section

### Crystal Structure Prediction

To investigate the stable cage‐like network configurations of the KC_12_ system under ambient pressure, this study employed crystal structure prediction methodology, conducting a global potential energy surface search using the CALYPSO^[^ ^28^ ^]^ software at 0 GPa. The unit cell volume was constrained to 250 Å^3^, with 1–3 formula units per composition considered. The maximum evolutionary generation was set to 30, generating 50 candidate structures per iteration. Energy minimization analysis revealed that the C_18_ cage‐like network configuration (KC_12_ stoichiometry) represents the lowest‐energy cage structure within the system.

### Electronic Structure Calculations

Structural optimization, isothermal‐isobaric ensemble (NPT ensemble), molecular dynamics (MD), and electronic structure calculations of carbon cages were carried out using the Vienna ab initio Simulation Package(VASP)^[^ ^67^, ^68^ ^]^ based on density functional theory. The calculations utilized projector augmented‐wave (PAW)^[^ ^69^ ^]^ pseudopotentials, with electronic wavefunctions expanded via a plane‐wave basis set. The exchange‐correlation interactions between electrons and ions were described using the Perdew‐Burke‐Ernzerhof (PBE) functional under the generalized gradient approximation (GGA).^[^ ^70^ ^]^ Full relaxation of atomic positions, lattice parameters, and unit cell volumes was performed during geometry optimization until atomic forces converged to 1 × 10^−7^ eVÅ^−1^. For structural optimization and electronic structure calculations, the cutoff energy was set to 600 eV, and K‐point grids of 9 × 9 × 12 were used in the Brillouin zone. The simulations were conducted at 1 atm to replicate ambient pressure. NPT‐MD simulations were carried out at 300 K and 1 atm with a supercell containing 324 atoms, using the Parinello‐Rahman algorithm to regulate temperature and pressure.

### Phonon Properties Calculations

Phonon frequencies and electron–phonon interactions for carbon cages were computed using the QUANTUM ESPRESSO^[^ ^71^, ^72^ ^]^ package. Wave function and charge density cutoff energies were set to 80 Ry and 600 Ry, respectively. Gaussian smearing was utilized to enhance convergence, with a smearing width of 0.02 Ry. The QE code employed a PAW‐type pseudopotential (*C*.*pbe* − *n* − *kjpaw*_−_ *psl*.1.0.0). In the Brillouin zone, 12 × 12 × 12 and 6 × 6 × 6 dense grids were used to calculate the Fermi surface and self‐consistent charge density, respectively. Phonon dynamical matrices and electron–phonon coupling matrix elements were computed on a 2 × 2 × 2 grid in the Brillouin zone using density functional perturbation theory and the Eliashberg equation. Fully relaxed lattice constants were used throughout the calculations.

McMillan derived an analytical expression for the semi‐empirical critical temperature by calculating the spectral function α^2^ *F*(ω) and solving the Eliashberg equation:^[^ ^73^, ^74^ ^]^

(1) $$T_{c}=\frac{\Theta_{D}}{1.45}exp\left[-\frac{1.04(1+\lambda )}{\lambda -\mu^{★}\left(1+0.62\lambda \right)}\right]$$

here, Θ_*D*_ is the Debye temperature; μ^⋆^ is the effective Coulomb pseudopotential, an empirical parameter typically chosen between 0.1 and 0.13; and λ is the electron–phonon coupling parameter, which directly reflects the strength of electron–phonon interactions. This formula was not applicable to systems with λ > 1. Therefore, Allen and Dynes^[^ ^45^ ^]^ modified this formula:

(2) $$T_{c}^{MAD}=\frac{\omega_{log}}{1.2}f_{1}f_{2}exp\left[-\frac{1.04\lambda }{\lambda -\mu^{★}\left(1+0.62\lambda \right)}\right]$$

In this equation, *f*_1_ and *f*_2_ are expressed as:

(3) $$f_{1}={\left[1+{\left[\frac{\lambda }{2.46(1+3.8\mu^{★})}\right]}^{3/2}\right]}^{1/3}$$

(4) $$f_{2}=1+\frac{\left(\omega_{2}/\omega_{log}-1\right)\lambda^{2}}{\lambda^{2}+{\left[1.82\left(1+6.3\mu^{★}\right)\left(\omega_{2}/\omega_{log}\right)\right]}^{2}}$$

*f*_1_ and *f*_2_ correspond to the strong‐coupling correction and the spectral shape correction, respectively. The revised McMillan formula is applicable to systems with 1.5 < λ < 3. For weaker electron–phonon coupling systems (λ < 1.5), *f*_1_ and *f*_2_ are assigned a value of 1, reducing the formula to:

(5) $$T_{c}^{MC}=\frac{\omega_{log}}{1.2}exp\left[-\frac{1.04\lambda }{\lambda -\mu^{★}\left(1+0.62\lambda \right)}\right]$$

Electron–phonon coupling parameter λ is derived from the integration of the Eliashberg spectral function α^2^ *F*(ω):

(6) $$\lambda =2\int_{0}^{\infty }\frac{\alpha^{2}F\left(\omega \right)}{\omega }d\omega$$

ω_*log*_ the logarithmic average of phonon frequencies, can be obtained directly from the phonon spectrum and is expressed as:

(7) $$\omega_{log}=exp\left[\frac{2}{\lambda }\int \frac{d\omega }{\omega }\alpha^{2}F\left(\omega \right)log_{\omega }\right]$$

## Conflict of Interest

The authors declare no conflict of interest.

## Supporting information

Supporting Information

## Data Availability

The data that support the findings of this study are available from the corresponding author upon reasonable request.
