# Supplementary material for: Superconducting Carbon‐Cage Network with T c of 109 K at Ambient Pressure
Source: Adv Sci (Weinh). 2025 May 29;12(30):e04281. doi: 10.1002/advs.202504281 (PMC12376711; doi:10.1002/advs.202504281)
Supplement: Supplementary file 1 — Supporting Information [file ADVS-12-e04281-s001.pdf]

ADVANCED SCIENCE
Open Access

Supporting Information

for *Adv. Sci.*, DOI 10.1002/advs.202504281

Superconducting Carbon-Cage Network with T_c of 109 K at Ambient Pressure

Z. F. Ye, David J. Singh, Y. N. Huang, Guo-Hua Zhong* and Hai-Qing Lin**

Supplementary information

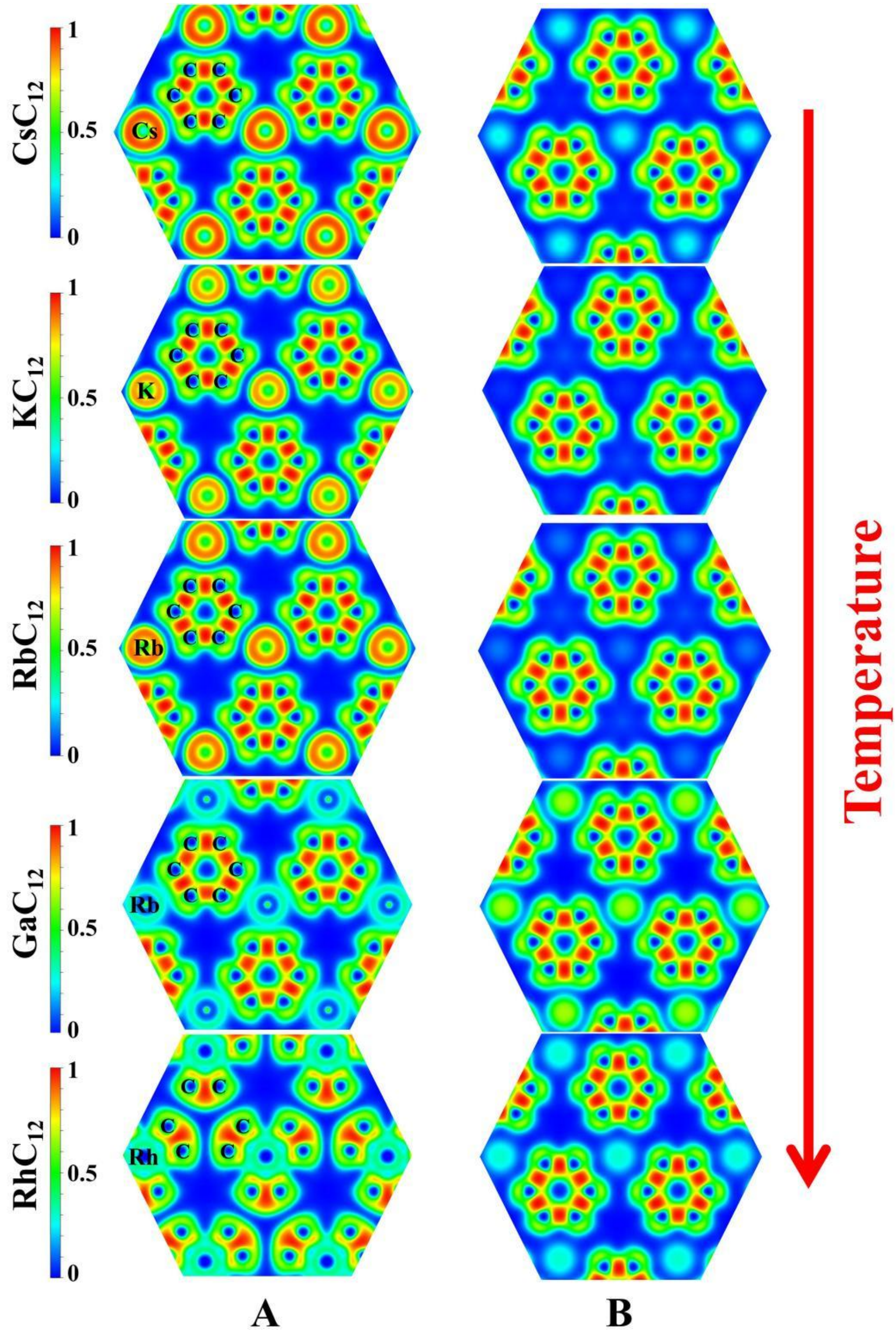

Fig. S1. Using the structural ELF, the bonding strength was analyzed, revealing stronger C-C covalent bonds in the MC_{12} cage network, which contributes to a higher transition temperature.

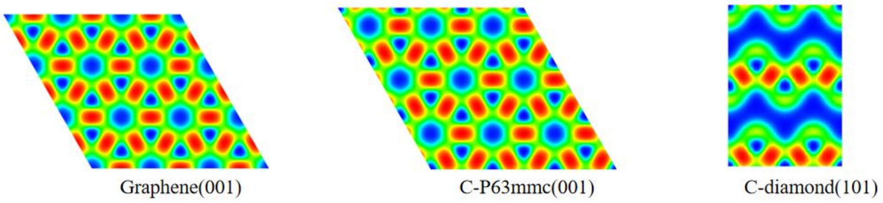

Fig. S2. ELF of graphene, C-P63mmc and diamond

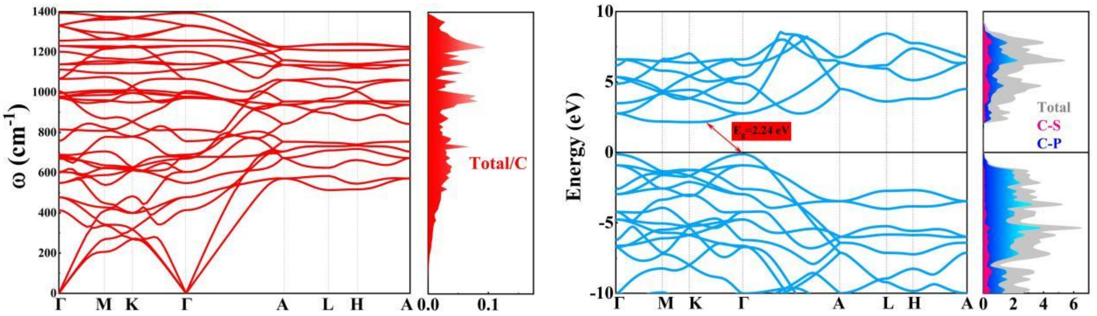

Fig. S3. The phonon spectrum, PhDOS, band structure, and DOS of the undoped C_{18} cage network.

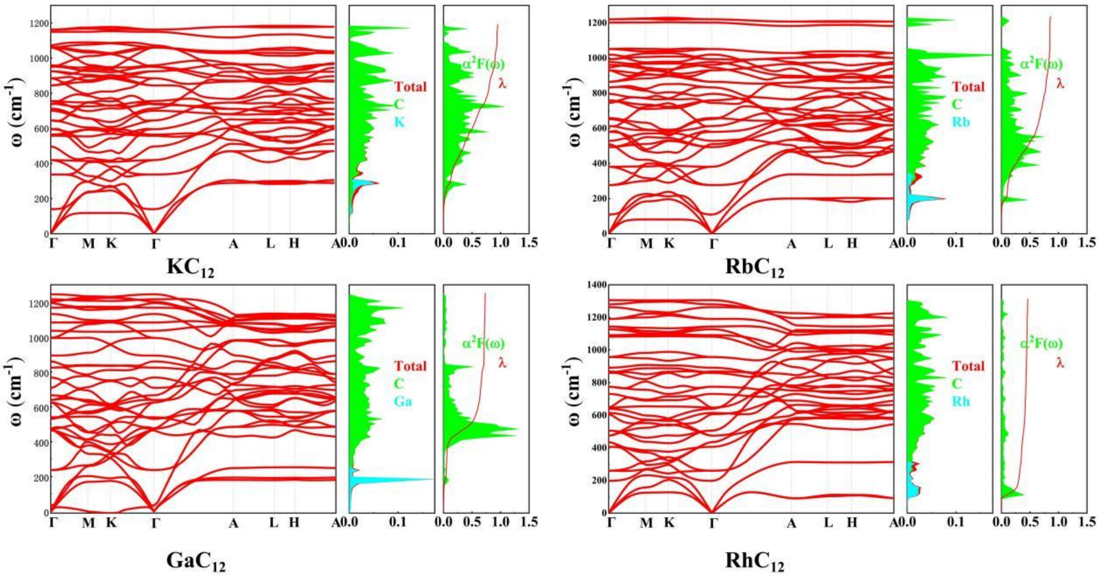

Fig. S4. The phonon spectra, PhDOS, Eliashberg spectral function, and electron-phonon coupling integrals of the MC_{12} cage network (M = K, Rb, Ga, and Rh).

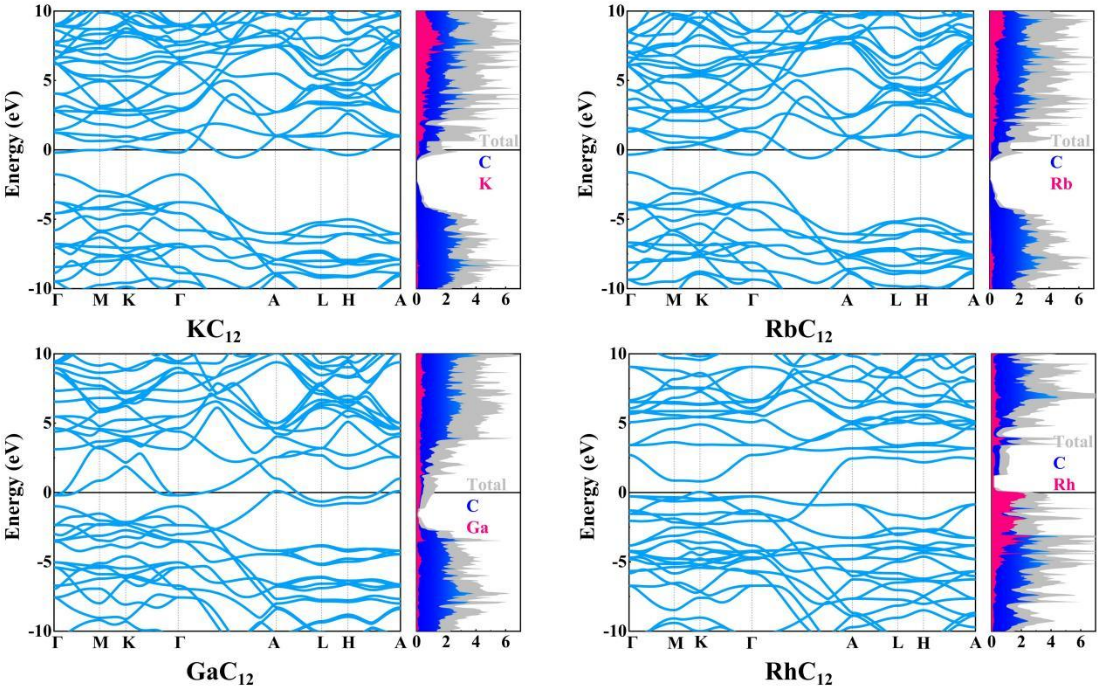

Fig. S5. The electronic band structures and DOS of the MC_{12} cage network (M = K, Rb, Ga, and Rh).

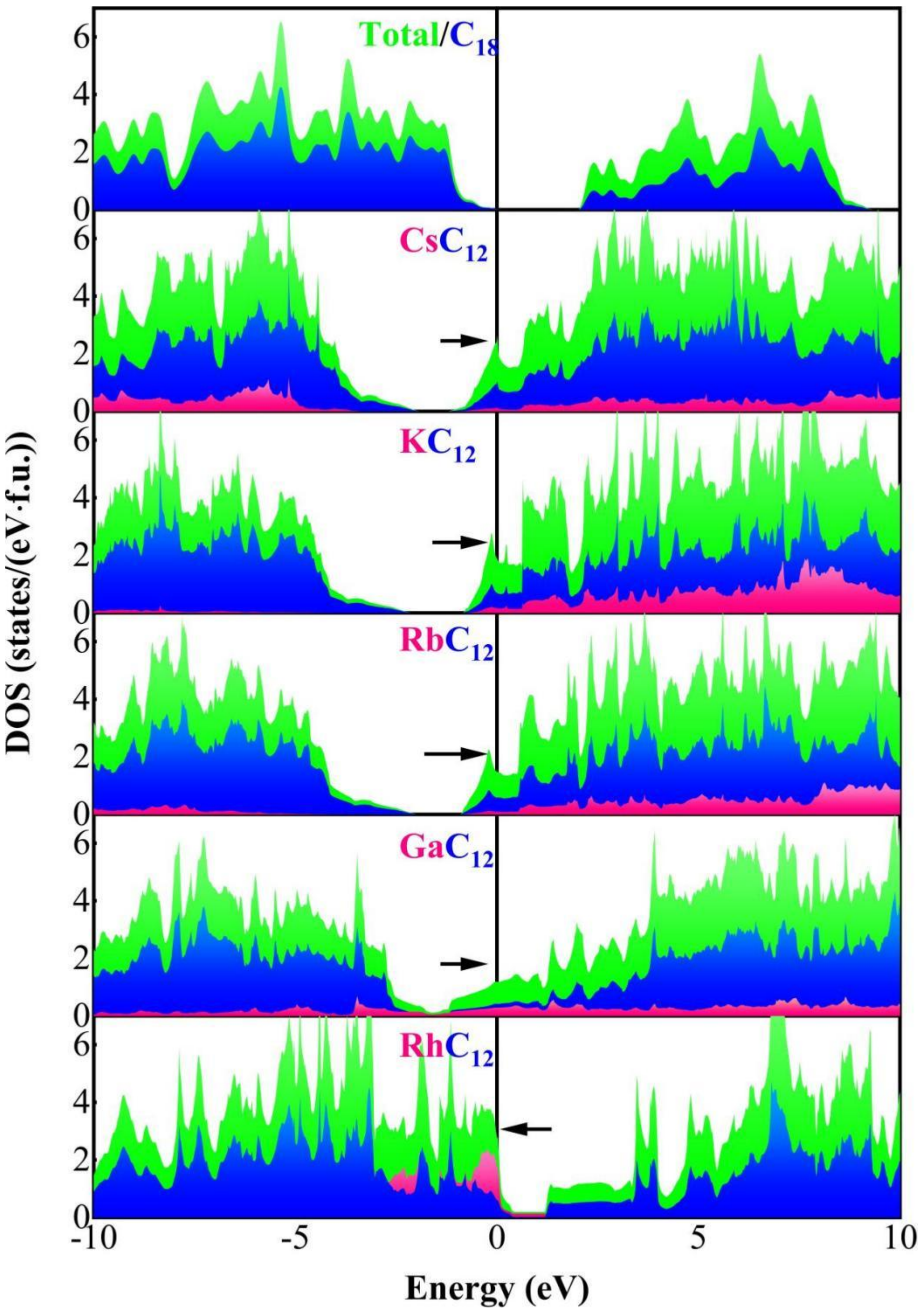

Fig. S6. Comparison of the DOS between element-doped C_{18} cage networks and the pristine structure.

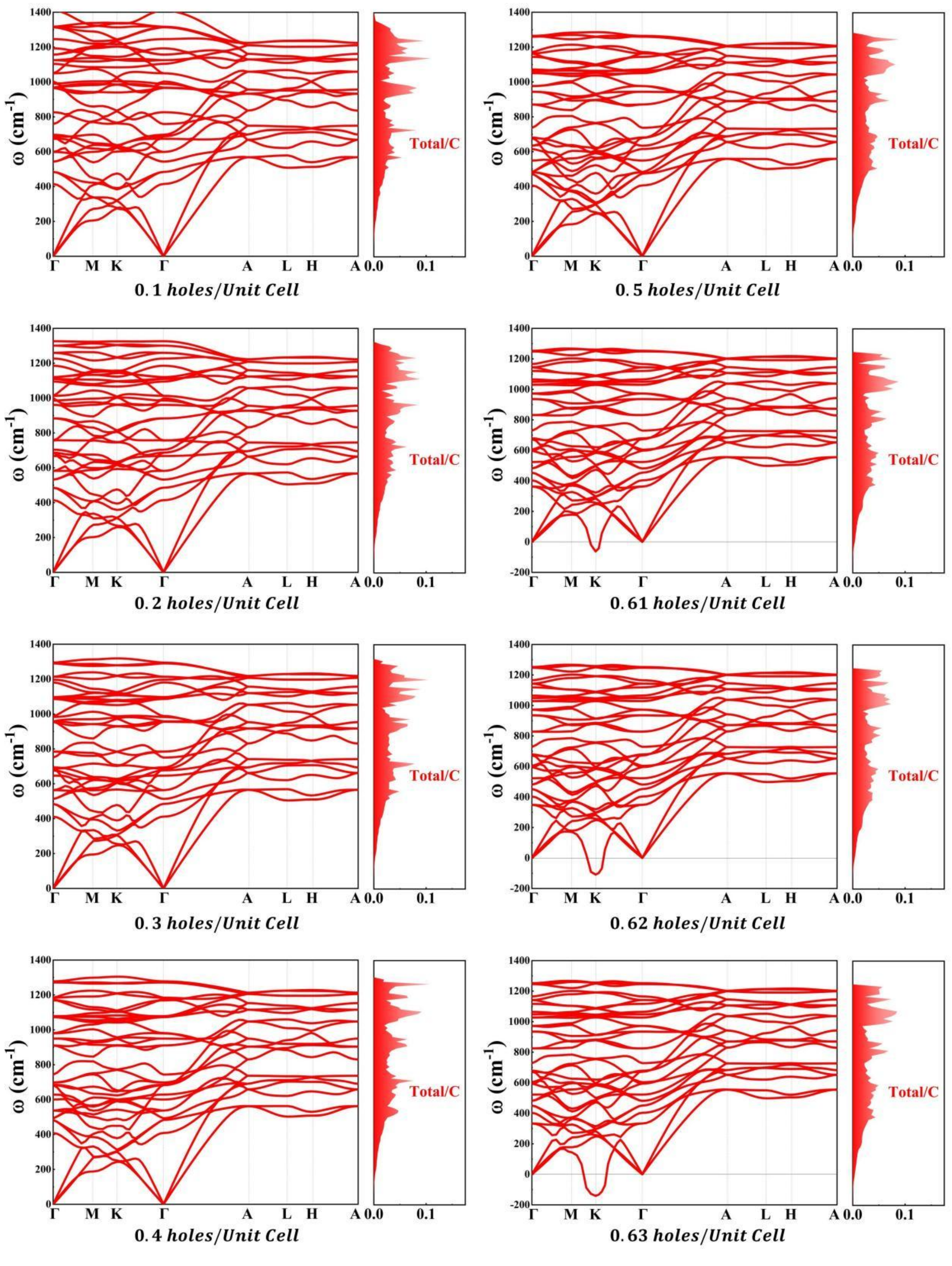

Fig. S7. The phonon spectra and PhDOS of C_{18} cage networks doped with varying hole concentrations.

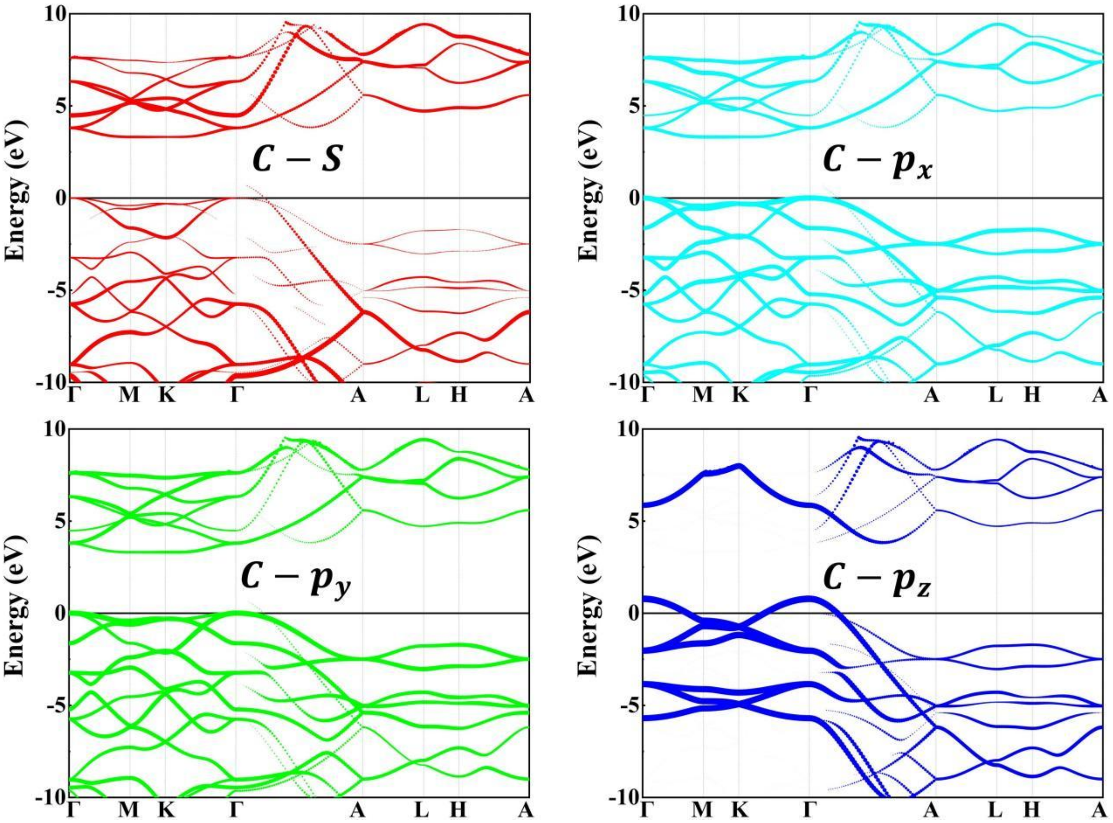

Fig. S8. The orbital-resolved band structure of C_{18} cage networks doped with 0.6 holes per unit cell.

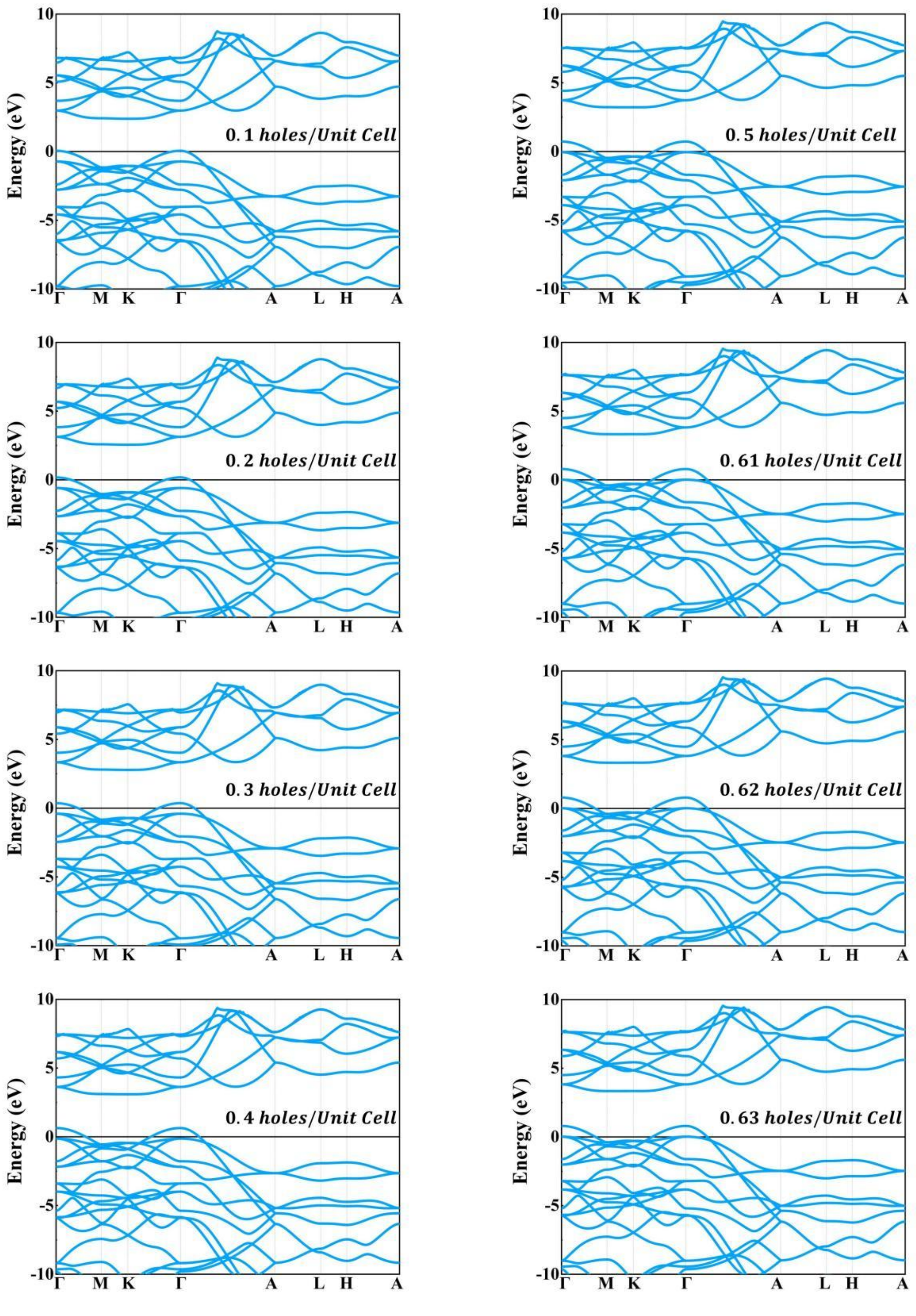

Fig. S9. The band structure of C_{18} cage network doped with different hole concentrations.

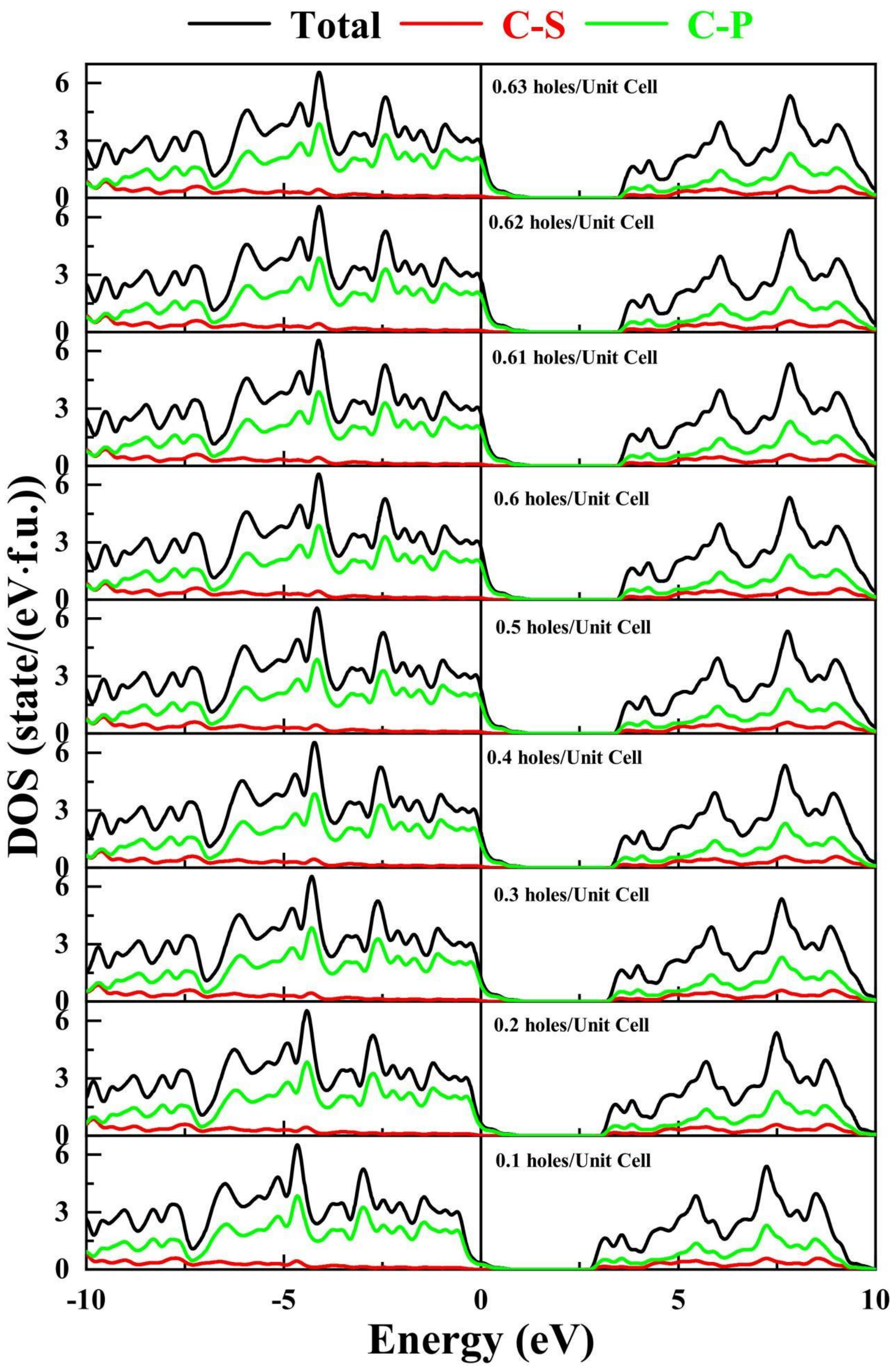

Fig. S10. The DOS of C_{18} cage network doped with different hole concentrations.

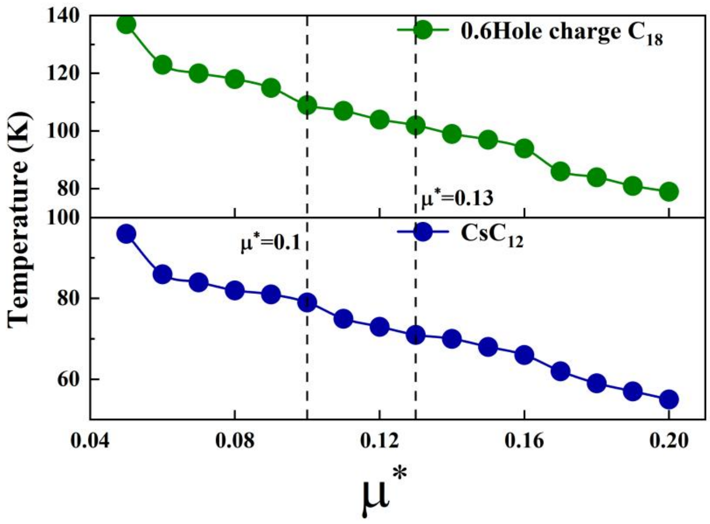

Fig. S11. Dependence of the superconducting transition temperature on μ*.

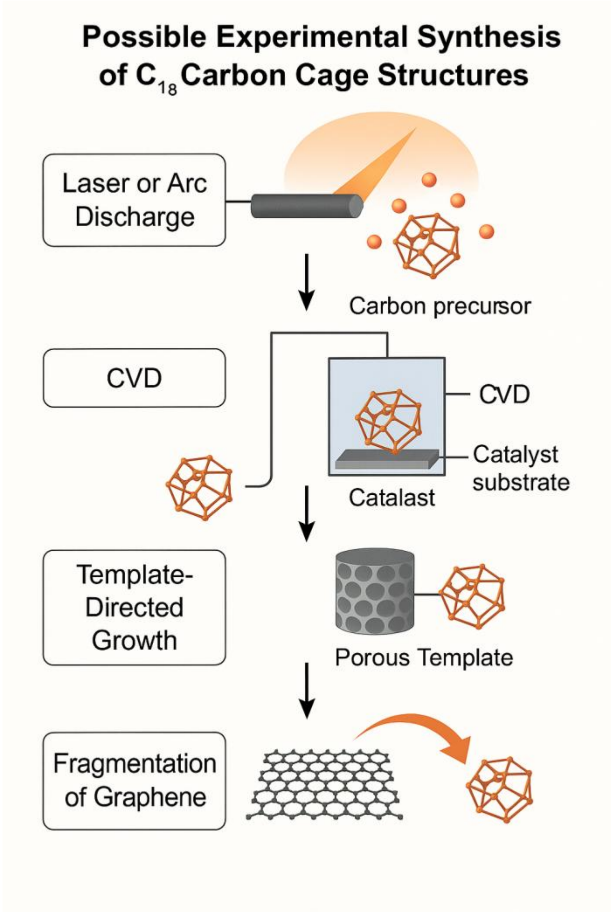

Fig. S12. Schematic of possible experimental synthesis routes for carbon cage structures

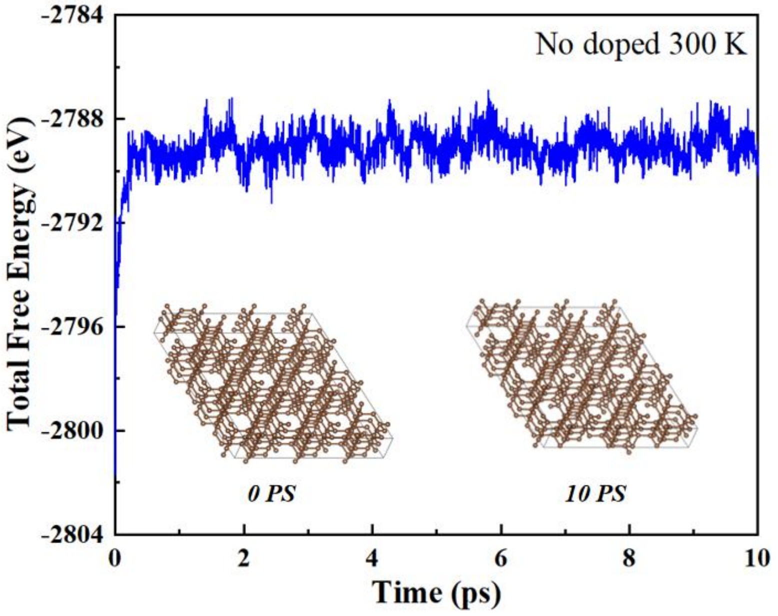

Fig. S13. AIMD simulation results of undoped C_{18} structure

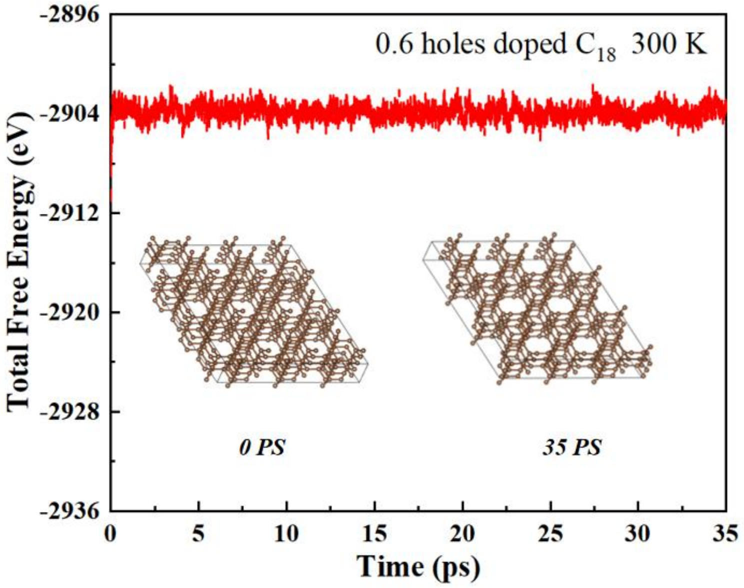

Fig. S14. AIMD simulation results of un0.6 holes doped C_{18} structure

TABLE. S1. Superconducting parameters of various clathrate structures at 0 GPa.

**Phase** $Amm2$	λ	ω_{log} (K)	N_{EF}	T_c (K)	
				$\mu^* = 0.1$	$\mu^* = 0.13$
CsC_{12}	1.9504	447.9	2.52	79.37	71.81
KC_{12}	0.9498	784.1	1.90	50.31	42.68

RbC_{12}	0.8565	670.5	1.49	35.90	29.63
GaC_{12}	0.7316	684.1	1.10	26.41	20.63
RhC_{12}	0.4595	285.9	2.79	2.43	1.33

Note: When lambda is greater than 1.5, the electroacoustic coupling is strong, and the McMillan Allen-Dynes modified formula is used to determine the superconducting transition temperature; when $\lambda \geq 1.5$, the simplified McMillan formula is used to determine the transition temperature.

TABLE. S2. Superconducting transition temperature corresponding to different hole concentrations in C_{18} cage network unit cell.

**Holes/Unit Cell**	$\boldsymbol{\lambda}$	$\boldsymbol{\omega_{log}}$ **(K)**	$\boldsymbol{N_{EF}}$	$\boldsymbol{T_c\ (K)}$	
				$\boldsymbol{\mu^* = 0.1}$	$\boldsymbol{\mu^* = 0.13}$
0.1	0.2298	1478.6	0.35	0.019	0.0
0.2	0.5530	1256.9	0.67	22.13	14.68
0.3	0.8565	1115.4	1.20	38.42	29.39
0.4	0.9549	982.3	1.83	63.59	54.02
0.5	1.2769	845.4	2.33	81.47	72.91
0.6	2.0112	611.1	2.68	109.32	102.06
0.61	2.3626	448.1	2.72	100.40	90.39
0.62	2.7616	346.0	2.74	59.94	56.84
0.63	4.0892	118.6	2.76	23.98	23.00

POSCAR file for MC_{12} cage network at 0 Gpa

Pure C_{18} cage network

```
1.0
        6.1250996590        -0.0000000000         0.0000000000
       -3.0625498295         5.3044919054         0.0000000000
        0.0000000000         0.0000000000         2.5509543419
    C
```

12
Direct

0.415189400	0.075368965	0.000000000
0.924631050	0.339830434	0.000000000
0.660169566	0.584810630	0.000000000
0.924631050	0.584810630	0.000000000
0.660169566	0.075368965	0.000000000
0.415189400	0.339830434	0.000000000
0.742039776	0.748521784	0.500000000
0.251478186	0.993517981	0.500000000
0.006482008	0.257960209	0.500000000
0.251478186	0.257960209	0.500000000
0.006482008	0.748521784	0.500000000
0.742039776	0.993517981	0.500000000

CsC_{12} cage network
1.0

6.5029811859	0.0000000000	0.0000000000
-3.2514905930	5.6317469073	0.0000000000
0.0000000000	0.0000000000	2.7360811234

C Cs
12 1
Direct

0.415946305	0.085329644	0.000000000
0.914670348	0.330626637	0.000000000
0.669373333	0.584053755	0.000000000
0.914670348	0.584053755	0.000000000
0.669373333	0.085329644	0.000000000
0.415946305	0.330626637	0.000000000
0.760788679	0.757218480	0.500000000
0.242781520	0.003570218	0.500000000
0.996429741	0.239211306	0.500000000
0.242781520	0.239211306	0.500000000
0.996429741	0.757218480	0.500000000
0.760788679	0.003570218	0.500000000
0.333330005	0.666670024	0.500000000

KC_{12} cage network
1.0

6.2743725777	0.0000000000	0.0000000000
-3.1371862888	5.4337660451	0.0000000000

0.0000000000 0.0000000000 2.6830008030
K C
1 12
Direct

0.333330005	0.666670024	0.500000000
0.415360004	0.079559997	0.000000000
0.920440018	0.335810006	0.000000000
0.664189994	0.584640026	0.000000000
0.920440018	0.584640026	0.000000000
0.664189994	0.079559997	0.000000000
0.415360004	0.335810006	0.000000000
0.753279984	0.752349973	0.500000000
0.247649997	0.000930000	0.500000000
0.999069989	0.246720001	0.500000000
0.247649997	0.246720001	0.500000000
0.999069989	0.752349973	0.500000000
0.753279984	0.000930000	0.500000000

RbC_{12} cage network
1.0

6.3993172646	0.0000000000	0.0000000000
-3.1996586323	5.5419713180	0.0000000000
0.0000000000	0.0000000000	2.6442780495

C Rb
12 1
Direct

0.417697102	0.083410755	0.000000000
0.916589260	0.334296346	0.000000000
0.665703654	0.582302928	0.000000000
0.916589260	0.582302928	0.000000000
0.665703654	0.083410755	0.000000000
0.417697102	0.334296346	0.000000000
0.759179771	0.757673740	0.500000000
0.242326260	0.001506018	0.500000000
0.998493969	0.240820244	0.500000000
0.242326260	0.240820244	0.500000000
0.998493969	0.757673740	0.500000000
0.759179771	0.001506018	0.500000000
0.333330005	0.666670024	0.500000000

GaC_{12} cage network
1.0

6.3361454010	0.0000000000	0.0000000000
-3.1680727005	5.4872628793	0.0000000000
0.0000000000	0.0000000000	2.5461442471

C Ga
12 1
Direct

0.416370422	0.078951269	0.000000000
0.921048760	0.337429136	0.000000000
0.662570834	0.583629608	0.000000000
0.921048760	0.583629608	0.000000000
0.662570834	0.078951269	0.000000000
0.416370422	0.337429136	0.000000000
0.740830183	0.747961342	0.500000000
0.252038658	0.992868841	0.500000000
0.007131123	0.259169787	0.500000000
0.252038658	0.259169787	0.500000000
0.007131123	0.747961342	0.500000000
0.740830183	0.992868841	0.500000000
0.333330005	0.666670024	0.500000000

RhC_{12} cage network

1.0

6.5375156403	0.0000000000	0.0000000000
-3.2687578201	5.6616546221	0.0000000000
0.0000000000	0.0000000000	2.5340156555

C Rh
12 1
Direct

0.409122705	0.083108947	0.000000000
0.916891098	0.326023757	0.000000000
0.673976243	0.590877295	0.000000000
0.916891098	0.590877295	0.000000000
0.673976243	0.083108947	0.000000000
0.409122705	0.326023757	0.000000000
0.667892754	0.721000373	0.500000000
0.278999597	0.946892381	0.500000000
0.053107608	0.332107216	0.500000000
0.278999597	0.332107216	0.500000000
0.053107608	0.721000373	0.500000000
0.667892754	0.946892381	0.500000000
0.333330005	0.666670024	0.500000000
